# Liver Ischemia After Transarterial Embolization for Hepatic Trauma Injury

**DOI:** 10.3390/diagnostics14222492

**Published:** 2024-11-07

**Authors:** Meng-Lin Han, Chia-Hsun Lu

**Affiliations:** 1 Department of Radiology, Wan Fang Hospital, Taipei Medical University, Taipei 116, Taiwan; 2 Department of Radiology, School of Medicine, College of Medicine, Taipei Medical University, Taipei 110, Taiwan

**Keywords:** hepatic injury, embolization, accessory right inferior hepatic veins

## Abstract

Transarterial embolization (TAE) is a routine procedure performed by interventional radiologists to manage traumatic hepatic injuries. Hepatic super-selective TAE rarely results in ischemia within the embolized area. In this case, the initial CT scan revealed significant contrast extravasation, leading to an urgent TAE. Due to continued concerns about bleeding, a second TAE was performed two days later. Subsequent CT scans revealed localized ischemia and necrosis following increased abdominal pain and elevated liver functions. Further analysis identified a common hepatic artery variation in this case. Thus, the ischemia in segments 6 and 7 was determined to be unrelated to the embolization procedure. A detailed review of the CT images suggests that injury to the accessory right inferior hepatic veins (IHVs) likely caused ischemia and eventual necrosis in segments 6 and 7. While hepatic vein variations are not uncommon, this case highlights the importance of evaluating the condition of hepatic veins, in addition to the hepatic artery and portal vein, during preoperative planning. If no arterial extravasation is identified, conservative treatment may be an option for the patient. Further research on this topic is warranted.

**Figure 1:**
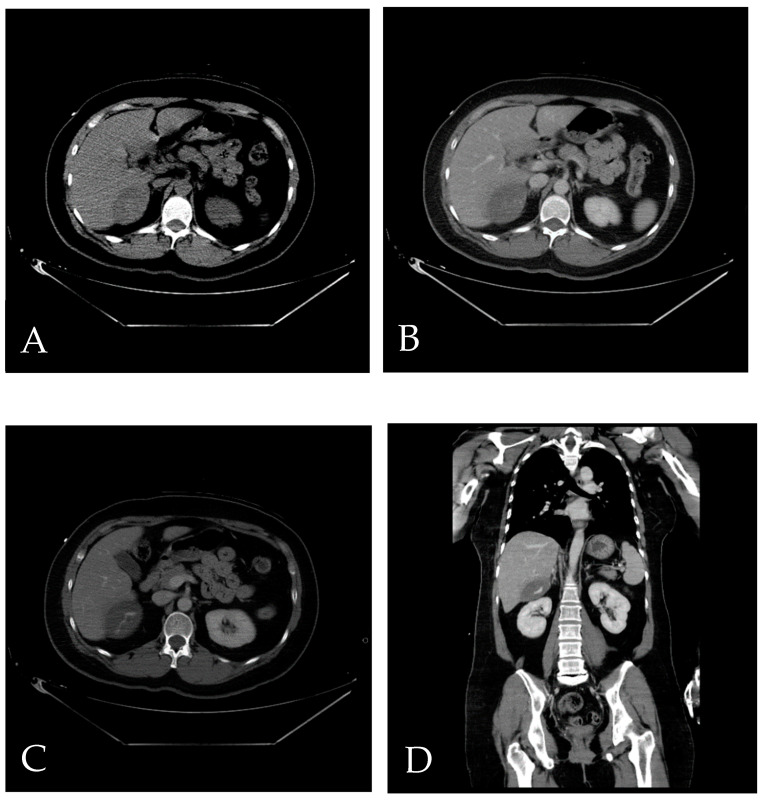
A 44-year-old woman indicated to further TAE was involved in a traffic accident [1]. (**A**) Axial-view CT without contrast reveals a well-defined lesion in the region of segments 6 (S6) and 7 (S7) of the liver, with a Hounsfield Unit (HU) value approximately around 60, indicative of a hematoma. (**B**,**C**) Axial view CT with contrast demonstrates contrast extravasation, highlighting the presence of vascular leakage. (**D**) The reconstructed coronal view CT with contrast provides a clearer visualization of the lesion, elucidating its precise location.

**Figure 2:**
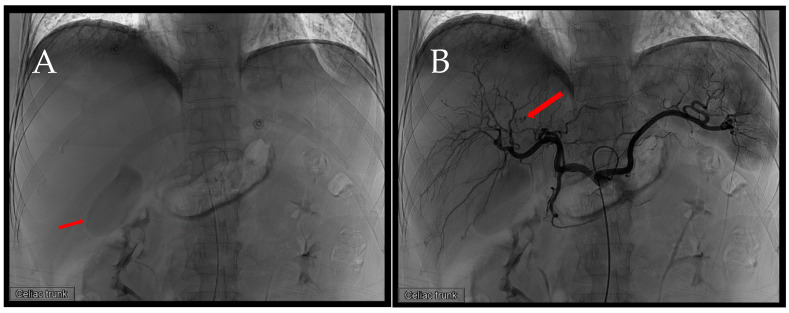
First TAE. After accessing the celiac trunk using a 4Fr. RH catheter, we performed a series of angiographies. (**A**) Prior to contrast injection, an evident hyperdense lesion (arrow) corresponding to the location identified in the CT scan is observed. The increased density is likely attributed to contrast extravasation during the preceding CT, thereby manifesting on this image. (**B**) A distinct extravasation (arrow) is visible, but its location does not align with the previously identified position in the CT scan.

**Figure 3:**
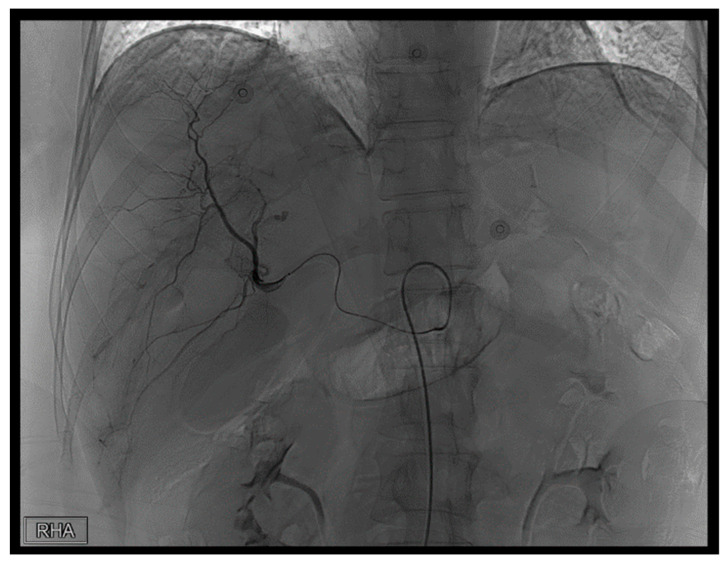
First TAE. In response to the previously identified extravasation, we utilized a 1.98Fr. Asahi microcatheter to access the right hepatic artery, followed by another angiography series. Similarly, the extravasation observed in the previous set of images is evident.

**Figure 4:**
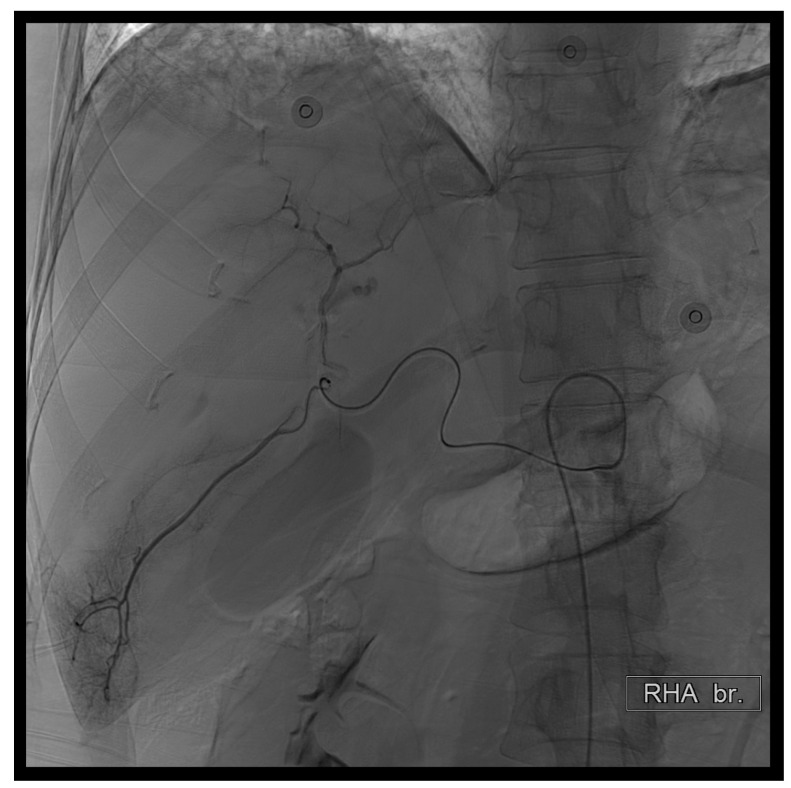
First TAE. Ultimately, due to patient restlessness, we advanced the microcatheter to the depicted position. Subsequently, we performed subselective embolization using a combination of 150–350 micrograms of EG gel diluted with contrast agent.

**Figure 5:**
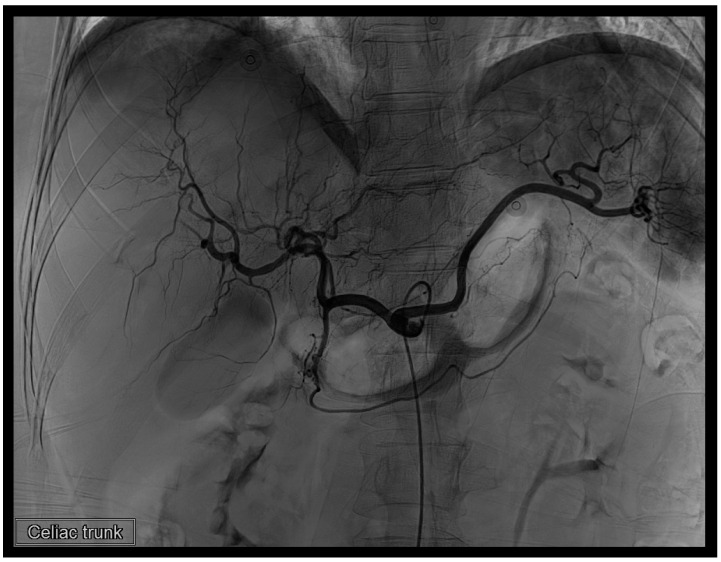
First TAE. Prior to concluding the procedure, we conducted another angiography at the celiac trunk. No evident extravasation was observed in this instance.

**Figure 6:**
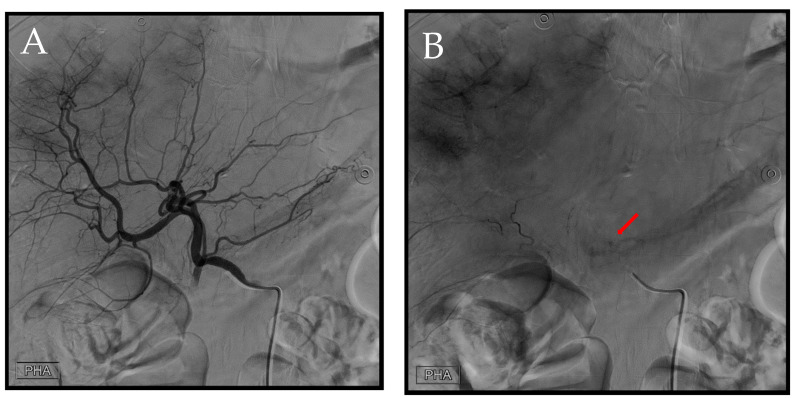
Representing the second transcatheter arterial embolization (TAE) session. (**A**) Angiography was performed using a 4Fr. RH catheter in the proper hepatic artery. (**B**) A distinct extravasation (arrow) was identified during this angiographic series.

**Figure 7:**
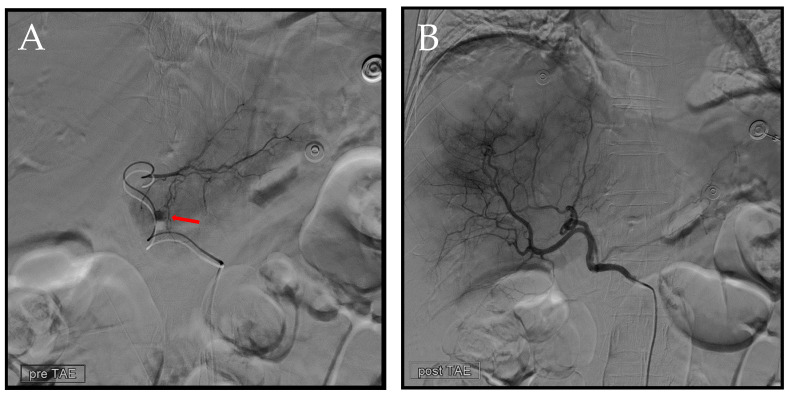
Second TAE. (**A**) Utilizing a 1.98Fr. Asahi microcatheter, access was established into a branch of the left hepatic artery. Subsequent angiography was performed to precisely identify the location of the extravasation (arrow). Following this, embolization was carried out using EG gel of a similar size to that used in the previous procedure. (**B**) Post-embolization, we conducted another angiography at the celiac trunk to confirm the absence of any additional extravasation.

**Figure 8:**
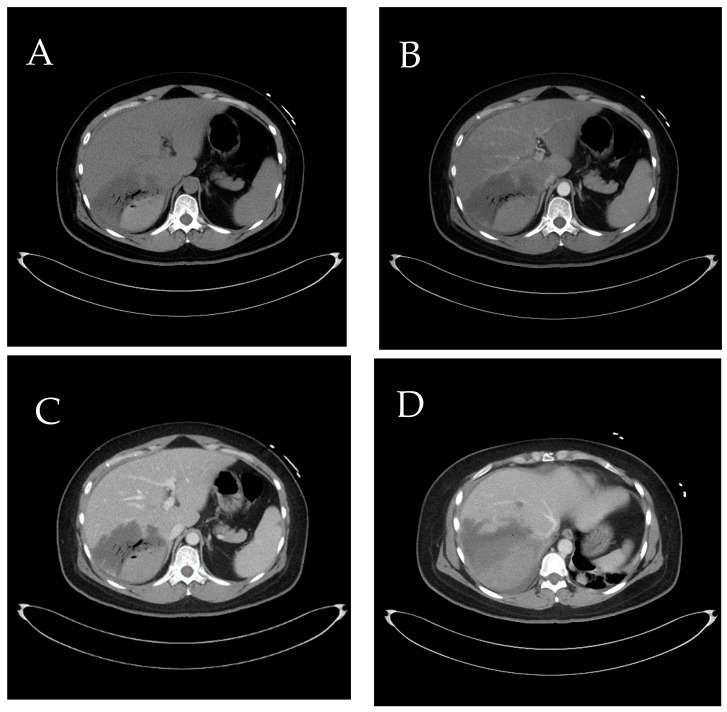
Four days following the initial TAE, a dynamic CT scan was conducted on the patient. (**A**–**C**) Representing the same anatomical level, precisely at the bifurcation height of the portal vein. (**A**) Pre-contrast phase. (**B**) Artery phase. (**C**) Portal phase. In comparison to the previous scans, an enlargement of the hematoma size is noticeable. Surrounding the hematoma, hypodensity and air are observed, suggesting necrotic changes induced by ischemia. (**D**) Depicting the bifurcation point of the left, middle, and right hepatic veins. The necrotic regions are still evident in this area.

**Figure 9:**
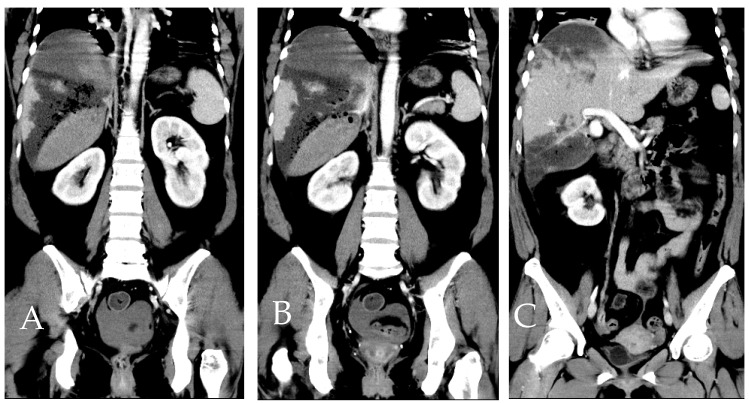
Dynamic CT scan in the artery phase, presented as a coronal view reconstruction. (**A**–**C**) Sections from ventral side (**A**) to caudal side (**C**) illustrate various planes of the patient. When correlated with the axial view from Figure 8, the approximate location of necrosis can be pinpointed to segments 6 (S6) and 7 (S7) of the liver that was not related to our embolization [2].

**Figure 10:**
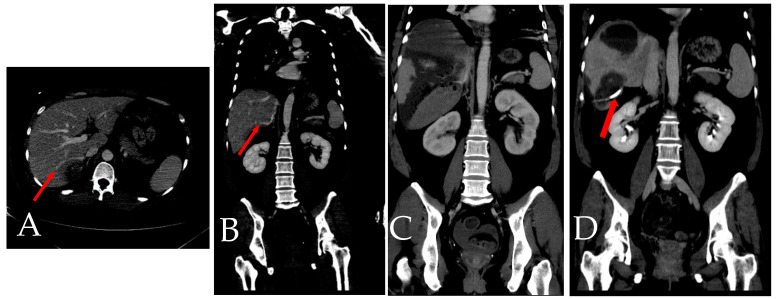
(**A**,**B**) Represent the initial CT scan, where the presence of accessory right inferior hepatic veins (IHVs) (arrow) is clearly visible from both axial and coronal views. These veins are notably in close proximity to the hematoma site. (**C**) Dynamic CT taken four days after the first transcatheter arterial embolization (TAE). At this point, the accessory right IHVs are challenging to trace and identify. (**D**) CT scan conducted three months later, and drainage tube is shown. The accessory right IHVs are no longer detectable, indicating a significant change or disappearance of these veins over time. Prior studies have shown that hepatic necrosis developed more commonly in cases with traumatic insult and nonselective proper hepatic artery embolization in the setting of patient decompensation or in cases with multiples sites of bleeding requiring numerous embolizations [3]. In this case, it is suggested that the injury to the right IHVs due to trauma may have led to the deterioration of the subcapsular hematoma [4]. This gradual increase in pressure subsequently resulted in congestion in dominant segments, ultimately causing ischemic necrosis.

## Data Availability

The data presented in this study are available upon request from the corresponding author.
